# Effect of circulating exosomes derived from normal-weight and obese women on gluconeogenesis, glycogenesis, lipogenesis and secretion of FGF21 and fetuin A in HepG2 cells

**DOI:** 10.1186/s13098-020-00540-4

**Published:** 2020-04-15

**Authors:** Reza Afrisham, Sahar Sadegh-Nejadi, Reza Meshkani, Solaleh Emamgholipour, Maliheh Paknejad

**Affiliations:** Department of Clinical Biochemistry, Faculty of Medicine, Tehran University of Medical Sciences, Tehran, Iran

**Keywords:** Exosome, Hepatokine, Insulin resistance, Obesity, Type 2 diabetes

## Abstract

**Background:**

It is generally accepted that obesity can lead to metabolic disorders such as NAFLD and insulin resistance. However, the underlying mechanism has been poorly understood. Moreover, there is evidence to support the possible role of exosomes in the metabolic homeostasis regulation. Accordingly, we aimed to determine the effect of plasma circulating exosomes derived from obese and normal-weight women on insulin signaling and the secretion of hepatokines in human liver cells.

**Methods:**

Plasma exosomes isolated from four obese (O-Exo) women and four normal-weight (N-Exo) female candidates were characterized for size, zeta potential, and CD63 protein expression and were used for stimulation of HepG2 cells. Then, cell viability, as well as levels of glycogen and triglyceride (TG), were evaluated. Levels of fetuin-A and FGF21 were measured using the ELISA kit. Expression of glucose 6-phosphatase (G6pase) and phosphoenolpyruvate carboxykinase (PEPCK) genes were determined using qRT-PCR. Western blot analysis was carried out to evaluating the phosphorylation of GSK3β.

**Results:**

The TG levels increased significantly in the cells treated with O-Exo than the control (vehicle) group (P = 0.005) and normal-weight group (P = 0.018). Levels of p-GSK3β and glycogen were significantly reduced by O-Exo in comparison with control (P = 0.002, P = 0.018, respectively). The mRNA expression of G6pase and PEPCK enzymes increased in the cells treated with O-Exo in comparison with the vehicle group (P = 0.017, P = 0.010, respectively). The levels of FGF21 in the supernatant of cells treated with O-Exo and N-Exo were significantly lower than the control group (P = 0.007).

**Conclusion:**

It appears that obesity-related circulating exosomes can impair insulin signaling pathways and associated components, increase intracellular TG content, and decrease FGF21 secretion in the hepatocytes.

## Background

Approximately 12% and 35% of the world’s population are obese and overweight, respectively [1]. It is generally accepted that obesity can lead to nonalcoholic fatty liver disease (NAFLD), insulin resistance and type 2 diabetes mellitus (T2DM) [1, 2]. A well-studied hypothesis by which obesity leads to initiation and development of the aforementioned conditions is that enlarged adipocytes secret the array of molecules such as pro-inflammatory adipocytokines may be detrimental to systemic insulin sensitivity [3]. Besides, free fatty acids (FFA) secreted from adipose tissue stimulate toll-like receptors (TLR) and leading to insulin resistance in liver and muscle cells [2, 4]. However, there is still a great deal of uncertainty in this regard.

More recently, the role of extracellular vehicles (EVs) in metabolic diseases has been received noticeable attention because these nano-sized vesicles act as a system carrying molecular and genetic information [5, 6]. In detail, EVs are released by different cells into body fluids such as plasma, cerebrospinal fluid, amniotic fluid, urine, aqueous humor and breast milk [7]. These spherical particles are stable in plasma [8] and play a key role in cell–cell communication as well as in biological functions and pleiotropic cellular [9–11]. Based on cellular origin and biogenesis, EVs are subdivided into exosomes, microvesicles, and apoptotic bodies [6, 7]. Lately, exosomes have garnered more attention as a key mediator of intercellular communication [12]. Exosomes are derived from the cell endosome and are in the range 50–200 nm [13]. Exosomes are consisting of lipids, proteins, genomic DNA, RNA and microRNA (miRNA) [7, 10, 11]. It has been established that there are some differences between the contents of exosomes derived from obese and normal-weight individuals especially in miRNA profiles [2, 12, 14] as well as it has been reported that adipose tissue acts as the main source of circulating exosomal miRNAs [13]. Nowadays, investigators have a particular interest in the EV research field. Indeed, the unique pattern of EVs and their cargo associated with obesity points toward possible role of EVs in the initiation and development of obesity and its metabolic complications.

More importantly, the current data provide information about the role of exosomes in the pathogenesis of NAFLD. Accordingly, the liver not only releases exosomes but also respond to exosomes/microvesicles secreted from other tissues. There is evidence that EVs secreted from different cell types send “pro-inflammatory/pro-fibrotic” signals to the liver [15, 16]. For instance, Koeck et al. observed that exosomes secreted from the visceral adipose tissues of obese patients cause impairment of the TGF-β pathway, which in turn leads to hepatic fibrosis [17]. In other studies, it was shown that circulating T cell-derived microvesicles from the blood of hepatitis patients induces nuclear factor kappa B up-regulation and fibrinolytic activation of hepatic stellate cells [18]. There are very few studies that investigate extracellular vesicles (such as exosomes) effects on insulin signaling [2, 19–21]. Deng et al. [21] reported for the first time that extracellular vesicles delivered from mice adipose tissue induced insulin resistance. Another study indicated that extracellular vesicles derived from obese subjects impaired insulin signaling in adipocytes [20]. Furthermore, it has been shown that Akt phosphorylation levels were reduced by extracellular vesicles derived from adipose tissue in hepatocytes and myocytes [2].

Today, several lines of evidence suggest the role of EVs in the metabolic homeostasis regulation, pathomechanism of obesity and its associated metabolic complications [5, 6]. There are well-documented studies that lipid accumulation, inflammation, and defective insulin signaling have a central role in the pathogenesis of NAFLD [22]. Although there is ample evidence on the close association between obesity and impaired insulin signaling, to the best of our knowledge, no study has investigated the effects of plasma circulating exosomes derived from obese and normal-weight females on insulin signaling pathways (such as glycogenesis and gluconeogenesis), lipogenesis and the secretion of hepatokines in human liver cells in vitro. Moreover, exact mechanisms linking obesity to metabolic disorders such as NAFLD and insulin resistance has been poorly understood. Therefore, in an attempt to unravel the underlying mechanism in which obesity could result in metabolic disorders such as NAFLD and insulin resistance, we aimed to determine the effect of circulating exosomes derived from obese and normal-weight women on insulin signaling and the secretion of hepatokines in human liver cells.

## Methods

### Study design and subjects

The current investigation was experimental in vitro study. A total of eight subjects including four obese women with body mass index (BMI) more than 30 kg/m^2^ and four normal-weight women with BMI between 20 and 24.9 kg/m^2^ were recruited for the current study. All subjects were selected among individuals who referred to Vesal blood transfusion center, Tehran province, Tehran, Iran during September 2018–December 2018 were selected. Individuals with a disease such as malignancies, coronary artery disease, osteoporosis, liver and renal dysfunctions, type 1 and 2 diabetes mellitus, myocardial infarction, systemic or local infections, autoimmune diseases, chronic or acute inflammatory disease, severe heart failure, and asthma as well as evidence of alcohol abuse and cigarette smoking were excluded. None of the subjects received medications such as anti-inflammatory drugs, immunosuppressive drugs, insulin therapy, vitamins as well as even antioxidant and micronutrient supplements during the previous 3 months.

### Sample and data collection

Initially, the characteristics of precipitants such as age, blood type, blood pressure, weight, and height were carefully obtained from subjects and then, BMI was calculated. The blood samples were collected from subjects. Different laboratory tests were applied to distinguish infections such as human immunodeficiency virus (HIV), hepatitis B, hepatitis C, and syphilis. After separating plasma, the samples were transferred to the Department of Clinical Biochemistry, Tehran University of Medical Sciences. The samples were stored at − 80 °C until the isolation of exosomes.

### Isolation of plasma exosomes

Plasma exosomes were isolated by ultracentrifugation as described previously [20]. Briefly, the plasma samples were diluted 1:3 volumes in sterile phosphate-buffered saline (PBS) and were centrifuged at 17,000×*g* for 30 min at 4 °C to remove cell debris. The supernatant was centrifuged at 100,000×*g* for 75 min at 4 °C using a Beckman L5-65 ultracentrifuge (Beckman Instruments, Palo Alto, CA, USA). The pellet was resuspended in PBS, filtered (0.22 μm) and then, centrifuged at 100,000×*g* for 75 min at 4 °C. The pellet (containing exosomes) was resuspended in PBS, aliquoted and kept at − 80 °C. The concentration of exosomes (according to their protein concentration) was measured by the Bradford method to co-incubation with HepG2 cells [23]. The hydrodynamic size and zeta potential of exosomes were determined using a Zetasizer Nano-ZS dynamic light scattering (DLS) measurement system (Malvern Zetasizer, ZEN3600, UK).

### Electron microscopic imaging of exosomes

Morphology and size of exosomes were determined using Transmission Electron Microscopy (TEM). Briefly, a drop of isolated exosomes (20 μL) was placed on 300 mesh carbon-coated TEM grid for 2 min, negatively stained with 2% aqueous uranyl acetate for 1 min. Then, the grid was examined on a Zeiss EM10C TEM operating at an accelerating voltage of 100 kV [24].

### Cell culture and stimulation with exosome and insulin

HepG2 cell lines were purchased from the Iranian Biological Resource Center (IBRC). The cells were maintained in Dulbecco’s Modified Eagle’s Medium (DMEM), supplemented with 1% penicillin–streptomycin solution, 10% fetal bovine serum (FBS) and then, incubated at 37 °C and 5% CO_2_. For treatments, the cells were washed with PBS and the cells were serum-starved for 12 h. Then, the cells were incubated inserum-free-DMEM supplemented with 4 µg/mL plasma exosomes derived from obese and normal-weight women, or vehicle (PBS). After 24 h incubation, HepG2 cells were induced with insulin (100 nM) for 15 min [2]. After washing again with PBS, the cells were collected for future examinations. All experiments were done on the same passage of cells and repeated three times for removing technical variables. The stimulation with insulin was only used for measuring glycogen levels, the phosphorylation of Glycogen synthase kinase 3 beta (GSK3β) and the mRNA expression of glucose 6-phosphatase (G6pase) and phosphoenolpyruvate carboxykinase (PEPCK) genes.

### Cell viability assay

The cytotoxicity of plasma exosomes against HepG2 cells was evaluated using the 3-(4,5-dimethyl-2-thiazolyl)-2,5-diphenyl-2*H*-tetrazolium bromide (MTT) method (Sigma). Briefly, the cells were seeded in 96-well plates and treated with the doses of 0.25–64 µg/mL the pooled exosomes obtained from obese women (PO-Exo). After 24 h of incubation at 37 °C, the media were removed and 100 μL MTT solution (0.5 mg/mL in PBS) was added to each well and incubated at 37 °C for 4 h. After removing the MTT solution, 100 μL dimethyl sulfoxide (DMSO) was added to each well and the plate was shaken in dark for 10 min. Finally, the absorbance was determined at 570 nm using the microplate reader (BioTek, USA). After the determination of the dose, the MTT assay was again used to evaluating the effect of plasma exosomes derived from normal-weight and obese females on cell viability.

### Measurement of triglyceride and glycogen

Glycogen levels were determined using the Glycogen Assay kit (Abcam Company, Cambridge, UK) following the manufacturer’s instructions. To the measurement of triglyceride (TG), the HepG2 cells were carefully washed four times with PBS and then, RIPA buffer was added to the cells on ice for 30 min. After ultrasonication, supernatant (50 µL) was applied for the measurement of protein using BCA assay. Next, a mixture of methanol and chloroform (1:2) were added to the rest of the supernatant on ice for 30 min and samples were centrifuged at 12,000×*g* for 5 min at 4 °C. After removing the upper solution, the lower solution was dried at 60 °C. Finally, 20 µL PBS was added to samples and vortexed for about one min [25]. TG levels were measured by kit based on the manufacturer’s instructions (Pars Azmoon Co., Tehran, Iran). The absorbance was measured at 570 and 546 nm for glycogen and TG assays using the ELISA reader (BioTek, USA), respectively.

### Oil red O staining

For oil red O staining, 5 × 10^5^ HepG2 cells were stained by the Oil Red O method to determine cellular lipid droplet accumulation. At first, HepG2 cells were washed several times with PBS and after fixation with 10% formalin, cells were stained with Oil Red O solution for 30 min at room temperature. Finally, the cells were washed four times with PBS and observed under a light microscope [26].

### Measurement of hepatokines

The enzyme-linked immunosorbent assay (ELISA) method was carried out to detect the protein levels of hepatokines in the supernatant of HepG2 cells. The levels of hepatokines [fibroblast growth factor 21 (FGF21) and fetuin-A] were measured using the ELISA kit for FGF21 (R&D Systems, Inc., Minneapolis, MN), and for fetuin-A (Abcam Company, Cambridge, UK) under the supplier’s instructions. The absorbance was measured at 450 nm using the ELISA reader (BioTek, USA), respectively.

### RNA extraction and quantitative real-time PCR

For evaluation of gluconeogenesis, gene expression of G6pase and PEPCK enzymes was determined using the quantitative real-time PCR (qRT-PCR). At first, RNA extraction was carried out using total RNA purification kit (Qiagen, GmbH, Hilden, Germany) and cDNA synthesis was performed using cDNA synthesis kit (TaKaRa Bio, Tokyo, Japan). Additional file 1: Table S1 showed the sequences of all used primers [27]. Gene expression of G6pase, PEPCK, and β-actin was determined by the qRT-PCR method using SYBR Green RealQ Plus Master Mix Green (Ampliqon, Skovlunde, Denmark) on the StepOnePlus Real-Time PCR System (Applied Biosystems, Foster City, USA). The mRNA expression of G6pase and PEPCK genes was normalized to β-actin expression. The delta–delta CT method was used to calculate the relative gene expression [28].

### Western blot analysis

The isolation of exosomes was approved by western blot analysis using the antibody of CD63 (Santa Cruz Biotechnology, Inc., Dallas, TX, USA). Also, the phosphorylation of GSK3β at Ser-9 position was determined in the cells treated with the doses of 2 and 4 µg/mL the pooled exosomes obtained from normal-weight (PN-Exo) females and PO-Exo as well as in the cells treated with the dose of 4 µg/mL obese exosome (O-Exo) and normal-weight exosome (N-Exo) using the western blot analysis. Briefly, the cells were lysed by RIPA buffer (50 mM Tris–HCl, 1% Triton X-100, pH 7.4, 0.2% SDS, 0.2% sodium deoxycholate, 1 mM PMSF and 1 mM Na-EDTA) containing protease inhibitor cocktail and then, the cell lysate was fractionated using SDS-PAGE and transferred to a polyvinylidene difluoride (PVDF) membrane. Five percent non-fat dry milk was applied to blocking. Immunoblots were incubated with antibodies against GAPDH, p-GSK3β, and GSK3β (Santa Cruz Biotechnology, Inc., Dallas, TX, USA) for 18 h, followed by incubation with horseradish peroxidase (HRP)-conjugated secondary antibodies for 60 min. The bound proteins were observed by chemiluminescence using enhanced electrochemiluminescence (ECL) reagents and subsequent autoradiography. Eventually, the protein bands were quantitated using ImageJ software.

### Statistical analysis

Descriptive statistics were applied to describe the participants’ characteristics. Continuous variables were evaluated for normality using the Shapiro–Wilk test. Not normally distributed data were logarithmically transformed. Normally distributed variables were considered as mean ± standard error of the mean (SEM) and differences between three and two groups were examined by one way ANOVA with Tukey HSD post hoc test and Student t-test, respectively. Not normally distributed variables were presented as the median and interquartile range (IQR) and differences between three and two groups were examined using the Kruskal–Wallis H and the Mann–Whitney U tests, respectively. The correlation between variables was measured by spearman correlation tests. *P*-value < 0.05 was considered significant. All analyses were carried out using IBM SPSS AMOS 21.0 (SPSS, Inc., Chicago, IL) and GraphPad Prism software, version 8 (GraphPad Software, La Jolla, CA).

## Results

### Participant characteristics

Normal-weight (n = 4) and obese (n = 4) women were recruited in our research. The mean age was 34.75 ± 3.09 years (± SD) for normal-weight subjects and 33.75 ± 3.20 years for obese subjects (P = 0.669). In addition, the median BMI was 23.85 ± 1.65 kg/m^2^ (± IQR) for normal-weight subjects and 34.30 ± 1.70 kg/m^2^ (± IQR) for obese subjects (P = 0.021). Participant characteristics are shown in Additional file 1: Table S2.

### Characterization of plasma exosomes

**Fig. 1:**
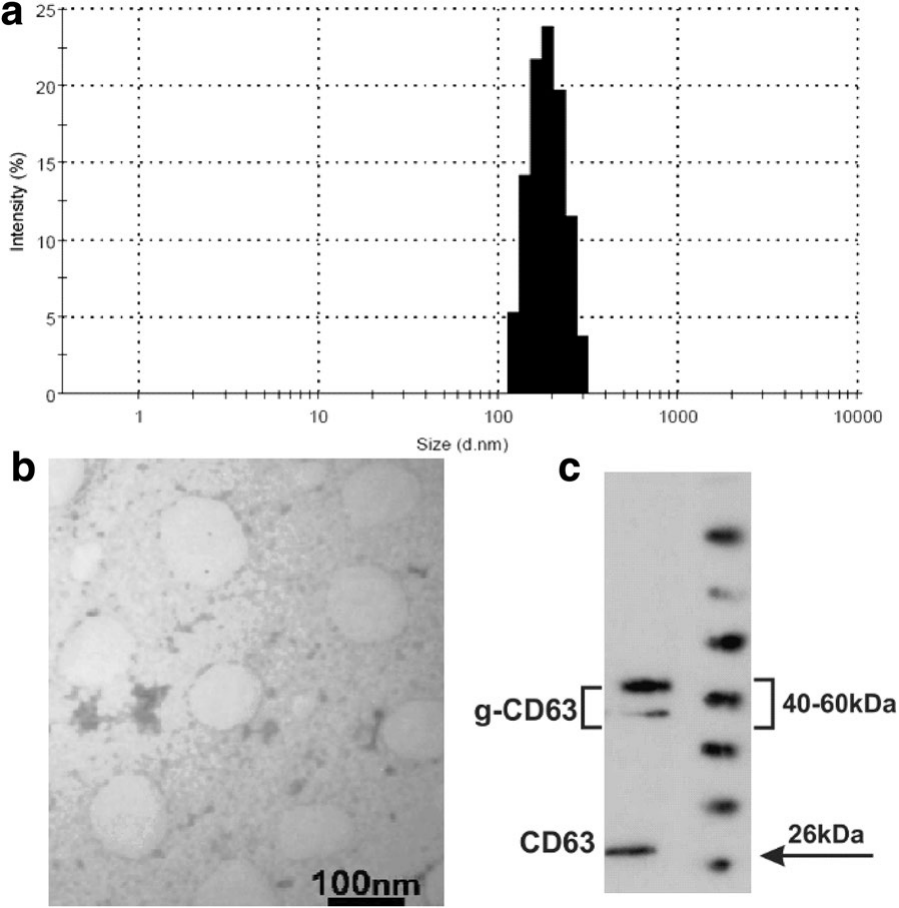
Characterization of plasma exosomes. The isolated exosomes were characterized for size, zeta potential and CD63 protein expression. **a** The mean hydrodynamic size of the isolated exosoms. **b** Morphology and size of isolated exosomes using TEM (scale bar, 100 nm). **c** Western blot results that confirmed the expression of common exosomal protein marker CD63

Figure 1 shows the characterization of plasma exosomes. The isolated exosomes were characterized for size, zeta potential, and CD63 protein expression. The mean hydrodynamic size of plasma exosomes was 161.1 nm (Fig. 1a) and the zeta potential of exosomes was − 4.01 mV at 25 °C. In addition, morphology and size of isolated exosomes were confirmed using TEM (Fig. 1b). As indicated in Fig. 1c, western blot results confirmed the expression of common exosomal protein marker CD63 in the sample.

### The determination of the dose

**Fig. 2:**
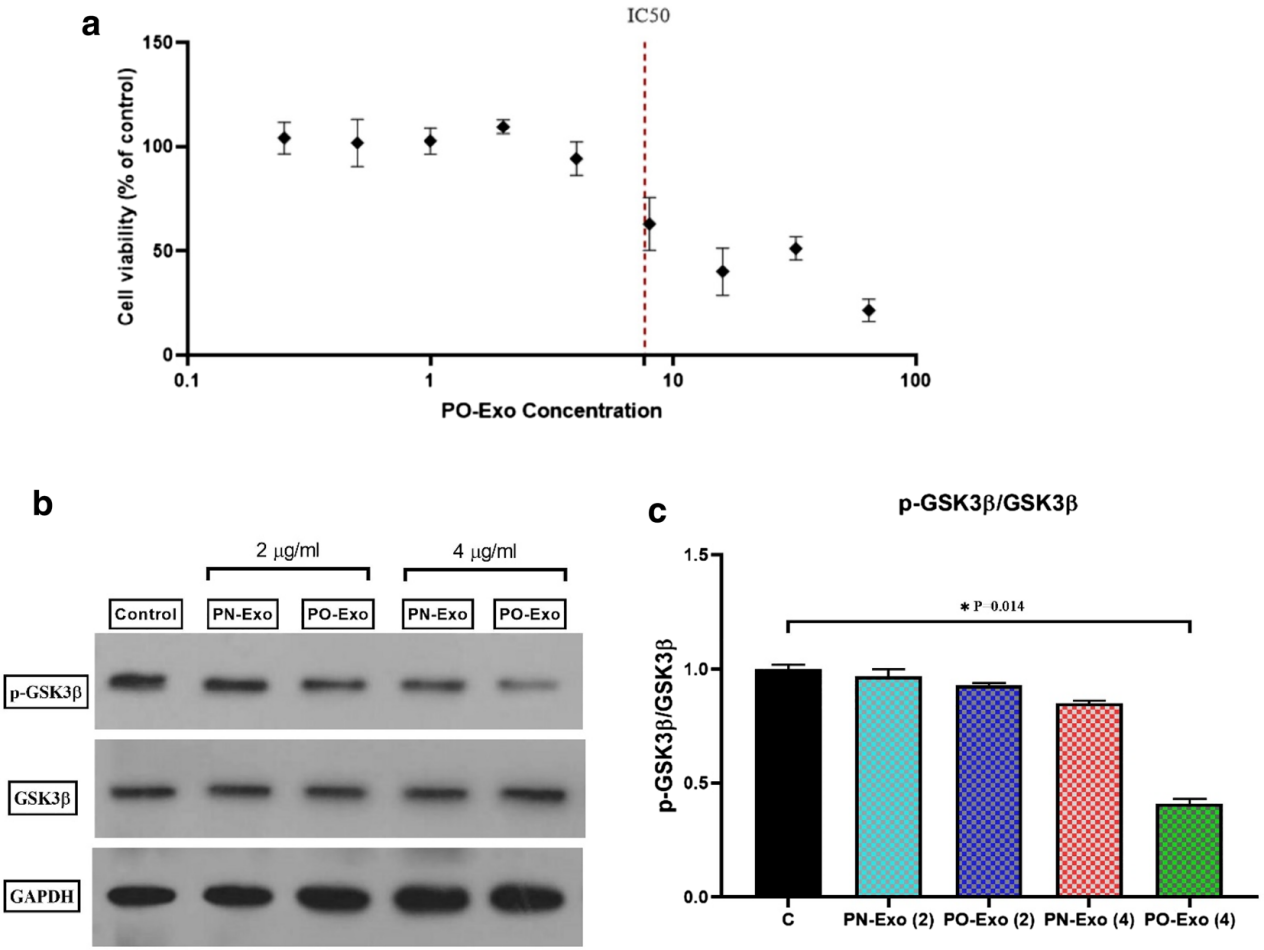
The different values of exosome were used for the determination of the dose. **a** The different doses of 0.25–64 µg/mL were applied in the MTT assay that the doses of 0.25–4 µg/mL were the optimal doses for the next experiments. **b** The phosphorylation of GSK3β at Ser-9 position was determined in the cells treated with the doses of 2 and 4 µg/mL PN-Exo and PO-Exo using western blot analysis that **c** based on p-GSK3β/GSK3β ratio, the dose of 4 µg/mL were detected as the optimal dose for the future treatments (P = 0.014). PN-Exo; pooled exosomes obtained from normal-weight women, PO-Exo; pooled exosomes obtained from obese women. The data are presented as median ± IQR

The different values of exosome were used for the determination of the dose using the MTT colorimetric test as well as the western blot method (Fig. 2). Based on Fig. 2a, the different doses of 0.25–64 µg/mL were applied in the MTT assay that the doses of 0.25–4 µg/mL were the optimal concentration for the next experiments. Then, the phosphorylation of GSK3β at Ser-9 position was determined in the cells treated with the doses of 2 and 4 µg/mL PN-Exo and PO-Exo using the western blot method (Fig. 2b) that based on p-GSK3β/GSK3β ratio, the dose of 4 µg/mL were detected as the optimal dose for the future treatments (P = 0.014, Fig. 2c).

### Effect of exosomes on cell viability and lipid contents

**Fig. 3:**
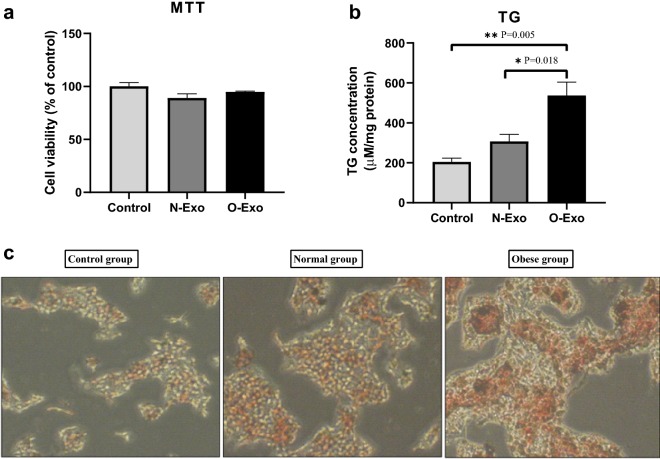
**a** MTT colorimetric test to measure cell viability following treatment with O-Exo, N-Exo and vehicle (PBS) as control group. **b** The effect of exosomes on intracellular triglyceride (TG) concentration in HepG2 cells. **c** Oil red O staining of HepG2 cells treated with vehicle (control group) and pooled exosomes obtained from normal-weight women (normal group) or obese women (obese group). The data are presented as mean ± SEM

The human liver cells were treated with PBS (control group), O-Exo and N-Exo. MTT colorimetric test was carried out to measure cell viability. As indicated in Fig. 3a, we found no significant differences between the three studied groups (P = 0.385). The TG levels in HepG2 cells treated with O-Exo increased significantly than the control (P = 0.005) and normal-weight (P = 0.018) groups (Fig. 3b). Moreover, the results of the Oil red O staining of the control group and pooled exosomes obtained from normal-weight women or obese women indicated that the lipid contents elevated in the obese group in comparison with other groups (Fig. 3c).

### Effect of exosomes on hepatic glycogenesis

**Fig. 4:**
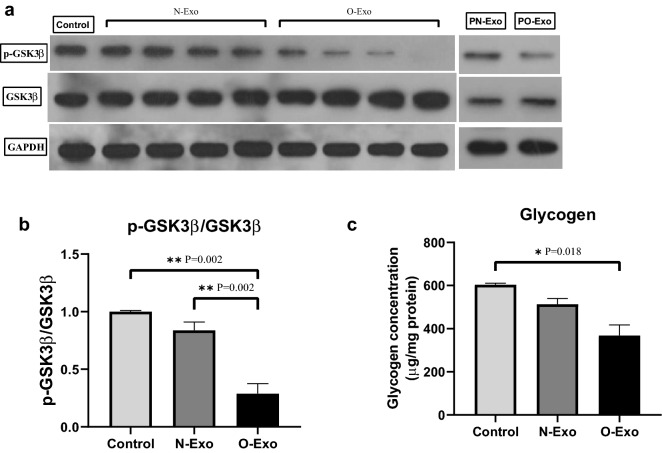
The effect of exosomes derived from normal-weight and obese women on **a** the phosphorylation of GSK3β using western blot analysis, **b** p-GSK3β/GSK3β ratio and **c** glycogen concentration. PN-Exo; pooled exosomes obtained from normal-weight women, PO-Exo; pooled exosomes obtained from obese women. The data are presented as mean ± SEM

After stimulation with exosome and insulin, phosphorylation of GSK3β at Ser-9 position was determined. We found significant differences between the three groups (P = 0.001). As indicated in Fig. 4a, b, p-GSK3β levels were significantly reduced by O-Exo in HepG2 cells in comparison with control (P = 0.002) and N-Exo groups (P = 0.002) but no significant difference was observed between control and normal-weight groups (P = 0.516). In line with this finding, we found a significant decrease in glycogen levels (P = 0.006) in cells treated with O-Exo than the control group (P = 0.018, Fig. 4c). As mentioned in Table 1, a strong positive correlation was detected between glycogen levels and p-GSK3β/GSK3β ratio (*r* 0.657, P = 0.020). Furthermore, p-GSK3β/GSK3β ratio and glycogen levels were negatively correlated with the levels of TG (*r* − 0.741, P = 0.006; *r* − 0.587, P = 0.045, respectively).

**Table 1:** The spearman correlation analysis between all variables

Variables	p-GSK3β/GSK3β	Glycogen	TG	PEPCK	G6pase	Fetuin A	FGF21
p-GSK3β/GSK3β							
*r*	1.000						
*P*-value							
Glycogen							
*r*	0.657	1.000					
*P*-value	0.020						
TG							
*r*	− 0.741	− 0.587	1.000				
*P*-value	0.006	0.045					
PEPCK							
*r*	− 0.783	− 0.427	0.706	1.000			
*P*-value	0.003	0.167	0.010				
G6pase							
*r*	− 0.804	− 0.552	0.650	0.937	1.000		
*P*-value	0.002	0.063	0.022	0.000			
Fetuin A							
*r*	0.490	0.462	− 0.469	− 0.434	− 0.427	1.000	
*P*-value	0.106	0.112	0.124	0.159	0.167		
FGF21							
*r*	0.545	0.462	− 0.413	− 0.587	− 0.636	0.588	1.000
*P*-value	0.067	0.112	0.183	0.045	0.026	0.035	

*Gly* glycogen, *TG* triglyceride, *PEPCK* phosphoenolpyruvate carboxykinase, *G6Pase* glucose 6-phosphatase, *FGF21* fibroblast growth factor 21, *p-GSK3β* phosphorylated-glycogen synthase kinase 3 beta

### Effect of exosomes on hepatic gluconeogenesis

**Fig. 5:**
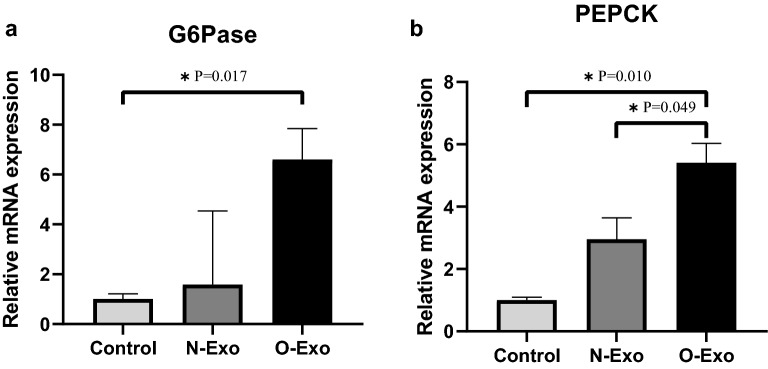
The effect of exosomes derived from normal-weight and obese women on **a** mRNA expression of G6Pase and **b** mRNA expression of PEPCK enzyme. The data related to PEPCK and G6Pase are presented as mean ± SEM and median ± IQR, respectively

We observed a significant up-regulation of G6pase and PEPCK enzymes among three groups (P = 0.016, P = 0.009, respectively). As demonstrated in Fig. 5a, the mRNA expression of G6Pase enzyme increased more than fivefold for HepG2 cells treated with O-Exo in comparison with control (P = 0.017), while, no significant difference was found between control and normal-weight group (P = 0.261). Regarding the mRNA expression of PEPCK enzyme, a significant difference was found between obese and control groups (P = 0.010) as well as between the cells treated with O-Exo and N-Exo (P = 0.049, Fig. 5b). According to the Table 1, a strong positive relation was the mRNA expression of G6Pase and PECPK enzymes (*r* 0.937, P < 0.001) that was also negatively correlated with p-GSK3β/GSK3β ratio (*r* − 0.804, P = 0.002, *r* − 0.783, P = 0.003, respectively). Furthermore, positive correlations were observed between the gene expression of gluconeogenesis enzymes and the TG levels (*r* 0.650, P = 0.022 for G6Pase, *r* 0.706, P = 0.010 for PEPCK).

### Effect of exosomes on hepatokines

**Fig. 6:**
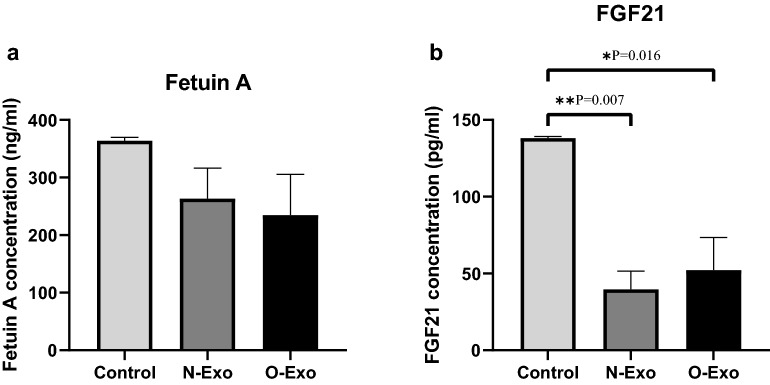
The effect of exosomes derived from normal-weight and obese women on **a** fetuin-A and **b** FGF21. The data are presented as mean ± SEM

As demonstrated in Fig. 6a, no significant difference was found between three groups for fetuin-A (P = 0.390). Notably, the levels of FGF21 in HepG2 cells treated with O-Exo and N-Exo were significantly lower than the control group (P = 0.007), however, no significant differences were observed between normal-weight and obese groups (P = 0.835, Fig. 6b). Based on Table 1, the levels of FGF21 were positively related to fetuin-A (*r* 0.588, P = 0.035). Moreover, negative correlations were identified between FGF21 protein levels and the gene expression of gluconeogenesis enzymes (*r* − 0.587, P = 0.045 for PEPCK, *r* − 0.636, P = 0.026 for G6Pase).

## Discussion

More recently, the importance of circulating exosomes as the possible communication mode among various tissues in the initiation and development of obesity-related metabolic disorders is starting to emerge from several studies [5, 6]. However, the underlying mechanism by which circulating exosomes connect obesity to metabolic diseases is still not fully understood. More importantly, we aimed to have a look at the effect of exosomes from obese and non-obese women to disclose the role of obesity in regulating insulin signaling and the secretion of hepatokines. To the best of our knowledge, this is the first study investigating the possible effect of plasma circulating exosomes from obese subjects on some components of insulin signaling pathways (such as glycogenesis and gluconeogenesis), the content of intracellular TG and the secretion of hapatokines in HepG2 cells.

In the current study for the first time, we have shown that the circulating exosomes derived from obese women can result in insulin signaling inhibition which in turn leads to hepatic insulin resistance. In detail, we found that exosomes from obese subjects significantly reduced both p-GSK3β/GSK3β ratio and glycogen levels in HepG2 cells in comparison with control. However, a noticeable increase in mRNA expression of G6pase and PEPCK enzymes was seen in the cells treated with O-Exo in comparison with untreated cells. These findings corroborate previous studies reporting the possible role of EVs (such as exosomes) effects on insulin signaling [2, 19–21]. For instance, EVs released from human adipose tissues dysregulated insulin signaling in hepatocytes which were shown by inhibiting insulin-induced Akt phosphorylation [2].

In line with our study, Deng et al. reported that EVs released from mice adipose tissue induced insulin resistance [21]. In another study, Mleczko et al. evaluated the effects of plasma EVs derived from obese and lean individuals on insulin responsiveness and glucose transport in cultured adipocytes. They showed that EVs derived from obese subjects reduced insulin-induced 2-deoxyglucose uptake in adipocytes. Also, they demonstrated that EVs obtained from hypoxic adipocytes reduced phosphorylation of AKT and impaired 2-deoxyglucose uptake [20]. Furthermore, Kranendonk et al. isolated EVs from human subcutaneous and omental adipose tissue and evaluated the effect of the EVs on insulin signaling in myocytes and hepatocytes. They showed that Akt phosphorylation levels were reduced by EVs derived from the majority of patients in hepatocytes. However, they observed the conflicting findings for the mRNA expression of G6pase and PEPCK enzymes in HepG2 cells and Akt phosphorylation levels in C2C12 cells. Since the EVs were obtained from patients with the different types of disease as well as the wide range of BMI levels; these conflicting results are justifiable [2]. While, in the present study, subjects without diseases were selected and subdivided into two groups (women with a BMI of more than 30 kg/m^2^ and women with BMI between 20 and 24.9 kg/m^2^). Then, the circulating exosomes were isolated from them. Accordingly, we found a noticeable increase in mRNA expression of G6pase and PEPCK enzymes in the cells treated with O-Exo in comparison with untreated cells.

Several studies have been reported that lipidosis in the myocytes [29, 30] and hepatocytes [31] is closely associated with the inhibition of insulin signaling. More importantly, we found that the triglyceride levels in the cells treated with O-Exo increased significantly in comparison with untreated cells and normal-weight groups. The excessive accumulation of triglycerides in hepatocytes in the form of intracellular lipid droplets is the hallmark of NAFLD. In line with our findings, Wang et al. reported that exosomes released by ductal epithelial cells and murine pancreatic cancer result in an increase of TG levels in murine skeletal muscle cells in a dose-dependent manner. They also indicated that the exosomes could inhibit glucose intake as well as insulin and PI3K/Akt signaling [19]. In another study, Koeck et al. investigated the effect of exosomes obtained from visceral adipose tissue from obese and lean patients on HepG2 cells. They showed that the exosomes could contribute to obesity-related liver disease possibly through dysregulation TGF-b pathway members [17].

According to our results and afore-mentioned evidence, it is tempting to speculate that the circulating exosomes obtained from obese subjects could interfere with hepatic insulin signaling pathways and contribute to hepatic insulin resistance and possibly consequent NAFLD.

Although the exact mechanisms by which plasma circulating exosomes from obese subjects could impair insulin signaling pathways and associated components cannot be ascertained based on our findings, several possibilities could be considered.

First, Kranendonk et al. [2] expressed that lower Akt phosphorylation in hepatocytes was related to monocyte chemoattractant protein-1 (MCP-1), IL-6, and macrophage migration inhibitory factor (MIF) concentrations in EVs that these factors might play a key role in obesity-induced insulin resistance [32]. Besides adipokines, EVs related FFAs might interfere with hepatocytes and lead to hepatic insulin resistance. FFAs are significant mediators in obesity-associated insulin resistance by elevating intracellular ceramides and diacylglycerol (DAG) [33, 34]. Moreover, it was shown exosomal microRNAs probably play important roles in insulin resistance. A variety of microRNAs are related to diabetic status and/or insulin resistance, such as miR-146a, miRs-375, 230d, 2130b-3p, 2374a-5p, 2423-5p, miRs-128, miR-103, and miR-223 [14].

The main aspect to consider is that our findings showed for the first time that circulating exosomes can decrease FGF21 secretion in the hepatocytes. FGF21, a member of the fibroblast growth factor family, is a well-known hepatokine involved in a variety of metabolic functions. There is evidence that this hepatokine can alleviate hepatic steatosis and insulin resistance. Specifically, FGF21 can inhibit lipid accumulation, enhance insulin sensitivity and lower body weight. It has been established that this hepatokine acts as a potential drug target for treating T2DM and even obesity [35, 36]. Alisi et al. [37] in 2013 showed that serum FGF21 was inversely related to hepatic damage in subjects with NAFLD. Studies have shown that FGF21-knockdown mice have remarkably exacerbated the accumulation of hepatic TG [38, 39] and overexpression of this hepatokine prevented the lipid accumulation [40]. On the other hand, Matikainen et al. [41] reported that FGF21 levels were negatively and positively correlated to postprandial TG and liver fat, respectively. Besides, in nonalcoholic steatohepatitis (NASH), serum FGF21 levels but not hepatic mRNA expression was increased [42]. However, it should be noted that evaluation of transcript levels of FGF21 in a dose- and time-manner dependent could explain how exosomes regulate different levels of gene expression. Hence, it is tempting to speculate that the reduction in secreted FGF21 in culture media from HepG2 cells may result indirectly from increased TG content in the cells. However, further assays are warranted to confirm this concept.

Putting these studies together, it seems that the current study adds new insight into the effect of obesity and related abnormalities on regulating insulin signaling and hepatokine secretion in liver cells. However, more complementary studies are needed to unravel the role of exosomes in the context of obesity in humans.

Although the current study along with others could partly open a new avenue toward the role of exosomes in the pathogenesis of metabolic disorders, the following limitation merits consideration. First, we did not investigate the content of exosomes (such as exosomal miRNAs/lipid/protein) and/or the effect of circulating exosome on other genes and proteins involved in insulin signaling pathways. Second, we did not confirm the effect of circulating exosomes derived from normal-weight and obese men on insulin signaling pathways in the HepG2 cell line. Moreover, further studies are required to determine the effects of obesity-related circulating exosomes on insulin signaling pathways in other cells such as primary hepatocytes, skeletal muscle cells, adipocytes, and immune cells.

## Conclusion

To the best of our knowledge, for the first time, we demonstrated that obesity-related circulating exosomes can impair insulin signaling pathways and associated components, as well as increase intracellular TG content, and decrease the secretion of FGF21 in the hepatocytes. Our study along with the previous data strengthens this concept that the plasma circulating exosomes derived from obese subjects play a possible role in obesity-related metabolic complications especially NAFLD possibly through inducing insulin resistance. However, more complementary studies are needed to unravel the role of exosomes in the context of obesity in humans.

## Supplementary information

**Additional file 1: Table S1.** The designed primers for qRT-PCR. **Table S2.** Demographics of obese and normal-weight women enrolled in the present study.

## Data Availability

The datasets used and/or analyzed during the current study available from the corresponding author on reasonable request.

## References

[1] Meex RC, Watt MJ (2017). Hepatokines: linking nonalcoholic fatty liver disease and insulin resistance. Nat Rev Endocrinol.

[2] Kranendonk ME, Visseren FL, van Herwaarden JA, Nolte-’t Hoen EN, de Jager W, Wauben MH (2014). Effect of extracellular vesicles of human adipose tissue on insulin signaling in liver and muscle cells. Obesity..

[3] Kahn BB, Flier JS (2000). Obesity and insulin resistance. J Clin Investig.

[4] Shi H, Kokoeva MV, Inouye K, Tzameli I, Yin H, Flier JS (2006). TLR4 links innate immunity and fatty acid–induced insulin resistance. J Clin Investig.

[5] Guay C, Regazzi R (2017). Exosomes as new players in metabolic organ cross-talk. Diabetes Obes Metab.

[6] Lawson C, Vicencio JM, Yellon DM, Davidson SM (2016). Microvesicles and exosomes: new players in metabolic and cardiovascular disease. J Endocrinol.

[7] Helwa I, Cai J, Drewry MD, Zimmerman A, Dinkins MB, Khaled ML (2017). A comparative study of serum exosome isolation using differential ultracentrifugation and three commercial reagents. PLoS ONE.

[8] Raposo G, Stoorvogel W (2013). Extracellular vesicles: exosomes, microvesicles, and friends. J Cell Biol.

[9] Ibrahim A, Marbán E (2016). Exosomes: fundamental biology and roles in cardiovascular physiology. Annu Rev Physiol.

[10] Dinkins MB, Enasko J, Hernandez C, Wang G, Kong J, Helwa I (2016). Neutral sphingomyelinase-2 deficiency ameliorates Alzheimer’s disease pathology and improves cognition in the 5XFAD mouse. J Neurosci.

[11] Van der Pol E, Böing AN, Harrison P, Sturk A, Nieuwland R (2012). Classification, functions, and clinical relevance of extracellular vesicles. Pharmacol Rev.

[12] Ferrante SC, Nadler EP, Pillai DK, Hubal MJ, Wang Z, Wang JM (2015). Adipocyte-derived exosomal miRNAs: a novel mechanism for obesity-related disease. Pediatr Res.

[13] Thomou T, Mori MA, Dreyfuss JM, Konishi M, Sakaguchi M, Wolfrum C (2017). Adipose-derived circulating miRNAs regulate gene expression in other tissues. Nature.

[14] Hubal MJ, Nadler EP, Ferrante SC, Barberio MD, Suh JH, Wang J (2017). Circulating adipocyte-derived exosomal MicroRNAs associated with decreased insulin resistance after gastric bypass. Obesity..

[15] Eguchi A, Feldstein AE (2018). Extracellular vesicles in non-alcoholic and alcoholic fatty liver diseases. Liver Res.

[16] Ban LA, Shackel NA, McLennan SV (2016). Extracellular vesicles: a new frontier in biomarker discovery for non-alcoholic fatty liver disease. Int J Mol Sci.

[17] Koeck ES, Iordanskaia T, Sevilla S, Ferrante SC, Hubal MJ, Freishtat RJ (2014). Adipocyte exosomes induce transforming growth factor beta pathway dysregulation in hepatocytes: a novel paradigm for obesity-related liver disease. J Surg Res.

[18] Kornek M, Popov Y, Libermann TA, Afdhal NH, Schuppan D (2011). Human T cell microparticles circulate in blood of hepatitis patients and induce fibrolytic activation of hepatic stellate cells. Hepatology.

[19] Wang L, Zhang B, Zheng W, Kang M, Chen Q, Qin W (2017). Exosomes derived from pancreatic cancer cells induce insulin resistance in C2C12 myotube cells through the PI3K/Akt/FoxO1 pathway. Sci Rep.

[20] Mleczko J, Ortega FJ, Falcon-Perez JM, Wabitsch M, Fernandez-Real JM, Mora S (2018). Extracellular vesicles from hypoxic adipocytes and obese subjects reduce insulin-stimulated glucose uptake. Mol Nutr Food Res.

[21] Deng Z-B, Poliakov A, Hardy RW, Clements R, Liu C, Liu Y (2009). Adipose tissue exosome-like vesicles mediate activation of macrophage-induced insulin resistance. Diabetes..

[22] Buzzetti E, Pinzani M, Tsochatzis EA (2016). The multiple-hit pathogenesis of non-alcoholic fatty liver disease (NAFLD). Metab Clin Exp.

[23] Razmkhah F, Soleimani M, Mehrabani D, Karimi MH, Kafi-Abad SA (2015). Leukemia cell microvesicles promote survival in umbilical cord blood hematopoietic stem cells. EXCLI J.

[24] Caradec J, Kharmate G, Hosseini-Beheshti E, Adomat H, Gleave M, Guns E (2014). Reproducibility and efficiency of serum-derived exosome extraction methods. Clin Biochem.

[25] Li X, Wang R, Zhou N, Wang X, Liu Q, Bai Y (2013). Quercetin improves insulin resistance and hepatic lipid accumulation in vitro in a NAFLD cell model. Biomed Rep.

[26] Kwon E-B, Kang M-J, Kim S-Y, Lee Y-M, Lee M-K, Yuk HJ (2018). Zanthoxylum ailanthoides suppresses oleic acid-induced lipid accumulation through an activation of LKB1/AMPK pathway in HepG2 cells. Evid Based Complement Altern Med..

[27] Luo Z, Zhang Y, Li F, He J, Ding H, Yan L (2009). Resistin induces insulin resistance by both AMPK-dependent and AMPK-independent mechanisms in HepG2 cells. Endocrine.

[28] Panahi G, Pasalar P, Zare M, Rizzuto R, Meshkani R (2018). High glucose induces inflammatory responses in HepG2 cells via the oxidative stress-mediated activation of NF-κB, and MAPK pathways in HepG2 cells. Arch Physiol Biochem..

[29] Virkamäki A, Korsheninnikova E, Seppälä-Lindroos A, Vehkavaara S, Goto T, Halavaara J (2001). Intramyocellular lipid is associated with resistance to in vivo insulin actions on glucose uptake, antilipolysis, and early insulin signaling pathways in human skeletal muscle. Diabetes.

[30] Kuhlmann J, Neumann-Haefelin C, Belz U, Kalisch J, Juretschke H-P, Stein M (2003). Intramyocellular lipid and insulin resistance: a longitudinal in vivo 1H-spectroscopic study in Zucker diabetic fatty rats. Diabetes.

[31] Ahmad Malik S, Acharya JD, Mehendale NK, Kamat SS, Ghaskadbi SS. Pterostilbene reverses palmitic acid mediated insulin resistance in HepG2 cells by reducing oxidative stress and triglyceride accumulation. Free Radic Res. 2019;1–13. 10.1080/10715762.2019.1635252 PMC6675602 31223033

[32] Kranendonk ME, Visseren FL, van Balkom BW, Nolte-’t Hoen EN, van Herwaarden JA, de Jager W (2014). Human adipocyte extracellular vesicles in reciprocal signaling between adipocytes and macrophages. Obesity..

[33] Wubbolts R, Leckie RS, Veenhuizen PT, Schwarzmann G, Möbius W, Hoernschemeyer J (2003). Proteomic and biochemical analyses of human B cell-derived exosomes potential implications for their function and multivesicular body formation. J Biol Chem.

[34] Laulagnier K, Motta C, Hamdi S, Sébastien R, Fauvelle F, Pageaux J-F (2004). Mast cell-and dendritic cell-derived exosomes display a specific lipid composition and an unusual membrane organization. Biochem J.

[35] Lin X, Liu YB, Hu H (2017). Metabolic role of fibroblast growth factor 21 in liver, adipose and nervous system tissues. Biomed Rep.

[36] Liu J, Xu Y, Hu Y, Wang G (2015). The role of fibroblast growth factor 21 in the pathogenesis of non-alcoholic fatty liver disease and implications for therapy. Metab Clin Exp.

[37] Alisi A, Ceccarelli S, Panera N, Prono F, Petrini S, De Stefanis C (2013). Association between serum atypical fibroblast growth factors 21 and 19 and pediatric nonalcoholic fatty liver disease. PLoS ONE.

[38] Fisher FM, Chui PC, Nasser IA, Popov Y, Cunniff JC, Lundasen T (2014). Fibroblast growth factor 21 limits lipotoxicity by promoting hepatic fatty acid activation in mice on methionine and choline-deficient diets. Gastroenterology..

[39] Tanaka N, Takahashi S, Zhang Y, Krausz KW, Smith PB, Patterson AD (2015). Role of fibroblast growth factor 21 in the early stage of NASH induced by methionine-and choline-deficient diet. Biochim Biophys Acta Mol Basis Dis.

[40] Chen S, Chen D, Yang H, Wang X, Wang J, Xu C (2020). Uric acid induced hepatocytes lipid accumulation through regulation of miR-149-5p/FGF21 axis. BMC Gastroenterol.

[41] Matikainen N, Taskinen M-R, Stennabb S, Lundbom N, Hakkarainen A, Vaaralahti K (2012). Decrease in circulating fibroblast growth factor 21 after an oral fat load is related to postprandial triglyceride-rich lipoproteins and liver fat. Eur J Endocrinol.

[42] Dushay J, Chui PC, Gopalakrishnan GS, Varela-Rey M, Crawley M, Fisher FM (2010). Increased fibroblast growth factor 21 in obesity and nonalcoholic fatty liver disease. Gastroenterology.

