# Supplementary material for: Effect of circulating exosomes derived from normal-weight and obese women on gluconeogenesis, glycogenesis, lipogenesis and secretion of FGF21 and fetuin A in HepG2 cells
Source: Diabetol Metab Syndr. 2020 Apr 15;12:32. doi: 10.1186/s13098-020-00540-4 (PMC7161281; doi:10.1186/s13098-020-00540-4)
Supplement: Supplementary file 1 — Additional file 1: Table S1. The designed primers for qRT-PCR. Table S2. Demographics of obese and normal-weight women enrolled in the present study. [file 13098_2020_540_MOESM1_ESM.docx]

**Table S1.** The designed primers for qRT-PCR

| **Gene** | **Forward/reverse primers** |
| --- | --- |
| G6Pase | F: 5^/^-CATTGACACCACACCCTTTGC-3^/^ |
|  | R: 5^/^-CCCTGTACATGCTGGAGTTGAG-3^/^ |
| PEPCK | F: 5^/^-GGCTACAACTTCGGCAAATACC-3^/^ |
|  | R: 5^/^-GGAAGATCTTGGGCAGTTTGG-3^/^ |
| β-actin | F: 5^/^-TCCTTCCTGGGCATGGAGT-3^/^ |
|  | R: 5^/^-ACTGTGTTGGCGTACAGGTC-3^/^ |

**Table S2.** Demographics of obese and normal-weight women enrolled in the present study

| **Subjects** | **Age (years)** | **BMI** |
| --- | --- | --- |
| **Obese_1_** | 31 | 33.4 |
| **Obese_2_** | 31 | 33.9 |
| **Obese_3_** | 37 | 34.7 |
| **Obese_4_** | 36 | 35.4 |
| **Normal_1_** | 35 | 22.0 |
| **Normal _2_** | 39 | 24.1 |
| **Normal _3_** | 33 | 24.0 |
| **Normal _4_** | 32 | 23.7 |
